# Factors Associated With Behavioral Intentions Toward Pain Assessment and Management Among Critical Care Nurses: A Theory‐Based Instrument Validation and Model Testing Study

**DOI:** 10.1155/jonm/7115245

**Published:** 2026-10-26

**Authors:** Zhi-Han Cai, Tsung-Cheng Hsieh, Li-Hwa Lin, Hui-Chun Chung, Sophia Huey-Lan Hu, Yi-Shan Hung, Yi-Ching Lin, Mei-Yen Lu, Chia-Hui Li, Ying-Shan Chen, Yuan-Mei Liao

**Affiliations:** ^1^ Department of Nursing College of Nursing National Yang Ming Chiao Tung University Taipei Taiwan nctu.edu.tw; ^2^ Department of Nursing College of Nursing Tzu Chi University Hualien Taiwan tcu.edu.tw; ^3^ Institute of Medical Sciences Tzu Chi University Hualien Taiwan tcu.edu.tw; ^4^ Taipei Municipal Gan-Dau Hospital (Managed By Taipei Veterans General Hospital) Taipei Taiwan; ^5^ Institute of Community Health Care College of Nursing National Yang Ming Chiao Tung University Taipei Taiwan nctu.edu.tw; ^6^ Department of Nursing Hualien Tzu Chi Hospital Buddhist Tzu Chi Medical Foundation Hualien Taiwan tzuchi.com.tw; ^7^ Department of Nursing Taichung Tzu Chi General Hospital Taichung Taiwan; ^8^ Department of Nursing Taitung MacKay Memorial Hospital Taitung Taiwan; ^9^ Department of Nursing Hsinchu Mackay Memorial Hospital Hsinchu Taiwan mmh.org.tw; ^10^ Department of Nursing and Department of Surgery Cardinal Tien Hospital New Taipei City Taiwan cth.org.tw; ^11^ Department of Nursing MacKay Memorial Hospital Taipei Taiwan mmh.org.tw; ^12^ Institute of Clinical Nursing College of Nursing National Yang Ming Chiao Tung University Taipei Taiwan nctu.edu.tw

**Keywords:** behavior, behavioral intention, critical care nurses, critically ill adults, intensive care unit, pain assessment and management, theory of planned behavior

## Abstract

**Background:**

Optimal pain management remains elusive despite the implementation of standard practice guidelines. The theory of planned behavior (TPB) offers a framework for understanding nurses’ clinical practices. Developing and validating theory‐based instruments enables an in‐depth exploration of the sociopsychological factors underlying nurses’ behavioral intentions and practices in pain assessment and management.

**Aims:**

This study aimed to develop and validate the Intention to Perform Pain Assessment and Management Questionnaire (IPPAMQ) and a Behavioral Scale, identify factors associated with nurses’ behavioral intentions, and evaluate the explanatory power of the TPB for nurses’ clinical practices.

**Methods:**

A two‐phase cross‐sectional study was conducted across seven institutions (December 2023–April 2025). Phase I (*n* = 200) employed a methodological design to establish the psychometric properties of the developed instruments. Phase II (*n* = 308) utilized the validated instruments to identify factors associated with nurses’ behavioral intentions and to evaluate the TPB’s explanatory power for nurses’ self‐reported practices through path analysis.

**Results:**

Both instruments demonstrated satisfactory psychometric properties. Nurses’ attitudes, subjective norms, and perceived behavioral control were significantly associated with their behavioral intentions, explaining 65% of the total variance, with perceived behavioral control being the strongest correlate. Nurses’ behavioral intentions were significantly associated with self‐reported practices, accounting for 47% of the variance and supporting the TPB’s substantial explanatory power for nurses’ practices.

**Conclusions:**

This study provides psychometrically robust instruments tailored to the TPB framework within the critical care context. The TPB framework effectively highlighted key sociopsychological correlates of nurses’ behavioral intentions and adequately accounted for nurses’ self‐reported pain assessment and management practices.

**Implications for Nursing Management:**

The IPPAMQ enables nurse managers and policymakers to conduct data‐driven evaluations of the sociopsychological factors and identify areas for tailored interventions. The TPB framework’s substantial explanatory power underscores the need for multilevel strategies to narrow the gap between nurses’ behavioral intentions and clinical practices.

## 1. Introduction

The pain experienced by critically ill adults in intensive care units (ICUs) may go unrecognized or undertreated [1]. Unrelieved pain impairs physiological and psychological well‐being [2, 3]. Previous studies have shown that approximately 53% of critical care nurses do not adequately follow standardized pain assessment and management protocols, and 38% of ICU patients undergo healthcare procedures without a documented pain assessment [4, 5]. Optimal pain management remains elusive despite the implementation of standard practice guidelines.

Nurses’ practices are crucial for optimal pain detection and management [1, 6]. While previous studies have focused on the relationships among nurses’ knowledge, attitudes, and practices [7–9], these factors do not fully explain the variability and complexity of nurses’ clinical practices. Furthermore, improving knowledge alone is insufficient to achieve sustained changes in nurses’ clinical practices [7, 10]. The theory of planned behavior (TPB) provides a comprehensive theoretical framework for examining the complexities of nurses’ healthcare behaviors. According to the TPB, sociopsychological constructs such as beliefs, attitudes, subjective norms (social pressure), and perceived behavioral control are crucial correlates underlying nurses’ healthcare behaviors.

Within the TPB, an individual’s behavioral intention is an immediate antecedent of behavior, which directly stems from three key constructs: attitude toward the behavior, subjective norm, and perceived behavioral control [11–14]. Previous studies have shown that nurses’ attitudes toward the behavior, subjective norms, and perceived behavioral control are correlated with their intentions to perform healthcare behaviors (e.g., using physical restraints), and that healthcare professionals’ behavioral intentions to carry out evidence‐based healthcare are associated with their actual implementation [15, 16]. However, intentions do not always translate into action. The explanatory power of intention depends on its strength and stability; more stable intentions lead to more consistent behavioral outcomes [17]. Therefore, strengthening and stabilizing behavioral intentions can maximize the likelihood of performing target behaviors.

Utilizing the TPB enables an in‐depth exploration of factors stemming from nurses themselves (e.g., attitudes) and the work environment (e.g., subjective norms) that underlie nurses’ behavioral intentions. This approach is valuable because attitude, subjective norm, and perceived behavioral control are theoretically modifiable targets for clinical improvement. Applying the TPB to the pain assessment and management practices of critical care nurses requires a psychometrically sound instrument. A valid and reliable instrument can measure nurses’ behavioral intentions and associated factors, explain variations in the adequacy of their practices, and aid in developing targeted interventions or educational programs to enhance behavioral intentions. We reviewed the literature on instruments measuring nurses’ pain assessment and management practices for adult patients, as well as those related to the TPB [8, 9, 18–21]. However, the psychometric properties of the identified instruments all require further evaluation. Instruments that simultaneously integrate the TPB’s main constructs within the context of adult critical care are lacking (Table S1).

This study had three aims. First, we developed the Intention to Perform Pain Assessment and Management Questionnaire (IPPAMQ) and a Behavioral Scale based on the TPB. We conducted comprehensive psychometric evaluations to address limitations in existing instruments and the lack of specialized instruments. These evaluations included expert content validation, item analysis, construct validity testing, and assessments of internal consistency and test–retest reliability to ensure the robustness of the developed instruments. Second, using the validated IPPAMQ, we aimed to identify factors associated with nurses’ behavioral intentions within the TPB framework. Third, we assessed the explanatory power of the TPB for nurses’ self‐reported pain assessment and management practices.

## 2. Methods

### 2.1. Study Design

This study had two phases. In Phase I, a methodological research design was used to develop and psychometrically validate the IPPAMQ and the Behavioral Scale. In Phase II, a cross‐sectional design was employed to identify factors associated with nurses’ behavioral intentions and to examine the explanatory power of the TPB for nurses’ self‐reported pain assessment and management practices.

### 2.2. Settings and Sample

In Taiwan, ICUs are predominantly located within medical centers or regional hospitals [22]. Thus, eligible sites for this study were Taiwan’s medical centers and regional hospitals. We recruited a convenience sample of nurses from the adult ICUs of two medical centers and five regional hospitals across northern, western, and eastern Taiwan. The inclusion criteria were age > 20 years, full‐time employment, and > 3 months of ICU experience. We excluded head nurses and assistant head nurses who spent limited time at the bedside.

The sample size was determined based on the requirements for the instrument’s psychometric validation in Phase I and the structural equation modeling (SEM) with path analysis in Phase II. For Phase I, a sample of 200 participants was targeted, as this is generally considered the minimum requirement for a confirmatory factor analysis (CFA) [23]. For Phase II, a minimum of 300 participants was required to achieve adequate statistical power for SEM with 7 or more constructs [24]. We approached 600 nurses and collected data from 508 across both phases. Additionally, 50 nurses from the Phase I sample completed the questionnaire twice to evaluate the test–retest reliability of the developed instruments.

### 2.3. Instrument

Data were collected using a questionnaire comprising three sections: individual characteristics, the IPPAMQ, and the Behavioral Scale. We used investigator‐developed items to collect individual characteristics: age, gender, educational level, institution type, ICU type, critical care experience, and training in pain assessment and management. The IPPAMQ and the Behavioral Scale used a seven‐point Likert scale [14] and were developed by a scale development committee comprising six members with expertise in critical care and scale development. This committee was responsible for generating operational definitions and items for constructs, reviewing and revising problematic items, and sending the revised items for further review and grading. Figure 1 presents a flowchart of the development and validation process for the developed instruments.

**FIGURE 1:**
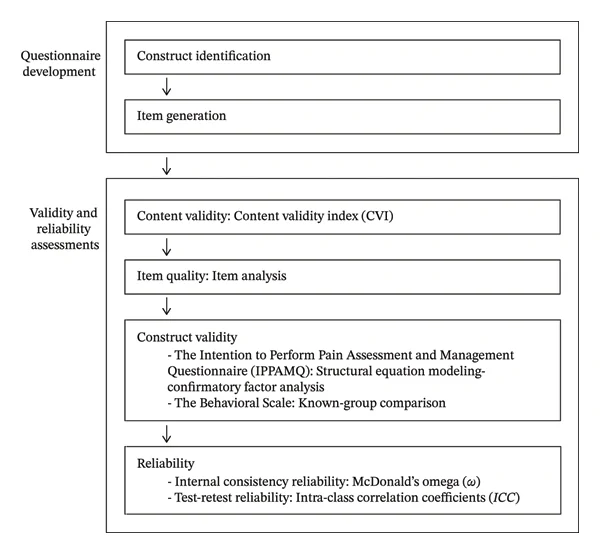
The development and validation process for the developed instruments.

#### 2.3.1. Item Generation and Content Validity

Within the context of critical care, the committee developed operational definitions for the TPB constructs (Table S2) and for pain assessment and management. For example, behavioral intention refers to the degree to which critical care nurses are prepared to adhere to updated practice guidelines in performing adequate pain assessment and management. Pain assessment and management are operationalized as interrelated components of nursing practice. Pain assessment involves systematically screening, quantifying, and documenting pain characteristics using standardized assessment tools. Pain management involves implementing pharmacological and non‐pharmacological interventions, along with regular re‐evaluations. Within the developed instruments, both components are examined simultaneously to reflect a continuous, integrated workflow in daily clinical practices, even though assessment and management may constitute distinct clinical tasks.

The committee members developed the items for the IPPAMQ and the Behavioral Scale based on the operational definitions and insights drawn from the literature relating to updated practice guidelines [1, 2], instruments measuring critical care nurses’ pain assessment and management [8, 9, 18], and publications relating to TPB questionnaires [14, 19–21]. The committee reviewed each item, deleted redundant words, and ensured that each item was clear and aligned with its corresponding construct. The committee members collaborated to finalize the items through eight intensive meetings.

Subsequently, we invited six experts with expertise in critical care and scale development to evaluate content validity. The six experts used two measures, adopting the content validity index (CVI) with a four‐point rating scale [25], to evaluate the developed items. The scores for semantic clarity ranged from “1 = not clear” to “4 = very clear and sufficient,” and for relevance to the corresponding construct ranged from “1 = not relevant” to “4 = relevant and sufficient.” The CVI values were calculated by dividing the number of experts who rated an item score of 3–4 by the total number of experts [26]. We deleted items that did not receive agreement from five experts and revised the remaining items based on their feedback. This grading, revision, and re‐grading process continued iteratively until all item CVI values were > 0.80 [25].

#### 2.3.2. The IPPAMQ

The IPPAMQ’s initial version comprises 38 items, with 5–6 per TPB construct. Seven subscale scores can be calculated by summing nurses’ response scores. A higher subscale score indicates greater intentions, more favorable beliefs and attitudes, stronger subjective norms, and greater perceived behavioral control. All items in this version demonstrated satisfactory content validity (CVI > 0.80).

#### 2.3.3. The Behavioral Scale

We developed a five‐item Behavioral Scale (Table S3) to assess the explanatory power of the TPB for nurses’ self‐reported pain assessment and management practices. The five items were developed to reflect core clinical healthcare behaviors in accordance with standard practice guidelines [1–3]. These items capture essential components of updated standard practices, including pre‐procedural pain assessment and management (Item 1), routine assessment, documentation, and management tailored to communication ability (Items 2 and 3), interprofessional communication during handovers (Item 4), and re‐evaluation for suboptimal pain control (Item 5). The Behavioral Scale scores are calculated by summing nurses’ responses; higher scores indicate more favorable pain assessment and management behaviors. The five items in this scale demonstrated satisfactory content validity (CVI = 1.00).

### 2.4. Data Collection

Data were collected between December 2023 and April 2025. Prior to the main study, a pilot study involving 10 critical care nurses was conducted. The pilot study participants reported no comprehension difficulties, with an average completion time of 15–20 min. The subsequent data collection was executed and partitioned into two phases for analysis. The first 200 responses were allocated to the Phase I sample for instrument validation. The subsequent 308 responses served as the Phase II sample for path analyses.

Two trained data collectors distributed informed consent forms and paper questionnaires and collected the completed materials after written consent was obtained. Some participants were unable to complete the questionnaire on the day it was distributed. They returned it within 20 days via a sealed collection box at the study sites. To assess the test–retest reliability, we invited 50 nurses to complete the questionnaires twice, with an interval of 10–14 days. Each participant received a gift (valued at ∼ USD 7) for participating.

### 2.5. Data Analysis

Data were analyzed using SPSS and AMOS version 24.0 (IBM Corp., Armonk, NY, USA). Statistical significance was set at *p* < 0.050 (two‐tailed).

#### 2.5.1. Phase I – Instrument Development and Validation

##### 2.5.1.1. Item Quality

Item quality was examined through item analysis, which involved assessing data normality (skewness < 3 and kurtosis < 8), item discrimination, and item‐total correlations [23, 24, 27]. We applied the following criteria: Cronbach’s *α* values of ≥ 0.70 after item deletion were deemed satisfactory; items were removed if the comparisons between the upper and lower 27% groups were not statistically significant; and the corrected item‐total correlation values were < 0.50 [24].

##### 2.5.1.2. Construct Validity

IPPAMQ: We examined the IPPAMQ’s construct validity using a CFA in the SEM procedure to verify its seven‐factor structure. The CFA employed maximum likelihood estimation, and model refinement involved retaining items with factor loadings ≥ 0.50, with model adjustments based on modification indices and standardized residuals > 4.00 [24]. Hair et al.‘s [24] recommendation regarding the application of model fit indices involves using at least one absolute fit index [e.g., Root Mean Square Error of Approximation (RMSEA) or Standardized Root Mean Residual (SRMR)] and at least one incremental fit index [e.g., Comparative Fit Index (CFI) or Tucker–Lewis Index (TLI)]. We used several model fit indices to evaluate the overall goodness‐of‐fit of the proposed models and used the Parsimony Normed Fit Index (PNFI) to assess a model’s parsimony. Recommended values for acceptable model fit are RMSEA ≤ 0.080, SRMR < 0.100 (values < 0.050 are considered better), CFI and TLI ≥ 0.900, and PNFI ≥ 0.500 [28, 29].

Convergent validity of the IPPAMQ was assessed by item factor loadings and the average variance extracted (AVE) and composite reliability (CR) of the subscales. We adopted the following evaluation criteria: standardized factor loadings ≥ 0.50, AVEs ≥ 0.50, and CRs ≥ 0.70 [24, 29]. To assess whether the seven subscales measure distinct constructs, the AVE value of each subscale was compared with the squared interfactor correlation values of each pair of subscales. Each subscale’s AVE value should be higher than the squared interfactor correlations between each pair of subscales [24, 30]. Inter‐factor correlations > 0.85 may indicate poor discriminant validity [31].

The Behavioral Scale: To examine the construct validity of the Behavioral Scale, we employed a known‐groups validation approach, comparing scores based on nurses’ training status (yes *vs.* no) and the ICU type in which they were employed (with *vs.* without surgical patients) using independent *t*‐tests. These two variables were selected for their theoretical and clinical links to nursing practice. Post‐licensure training directly enhances nurses’ adherence to standard practice protocols, thereby fostering more frequent and adequate pain assessment and management [32]. Critical care settings admitting surgical patients routinely expose nurses to a high volume of acute, observable postoperative pain scenarios. This persistent clinical exposure, coupled with structured protocols, reinforces proactive pain assessment and management [33]. Therefore, trained nurses and those working in the ICUs caring for surgical patients were hypothesized to exhibit higher Behavioral Scale scores, thereby justifying the use of these distinct groups to confirm the scale’s construct validity.

##### 2.5.1.3. Reliability

Reliability testing included internal consistency and test–retest reliability. Internal consistency was evaluated using McDonald’s Omega (*ω*), which overcomes the limitations of Cronbach’s *α* by allowing for varying factor loadings within a construct rather than assuming equal loadings [34]. Internal consistency reliabilities ≥ 0.70 are considered acceptable [23, 34]. The test–retest reliability was assessed using the intraclass correlation coefficient (*ICC*), based on a two‐way mixed model. The *ICC* was evaluated as 0.40–0.59 (fair), 0.60–0.74 (good), and ≥ 0.75 (excellent) [35].

#### 2.5.2. Phase II—Evaluation of Model Explanatory Power

Path analyses were conducted to examine the structural relationships among TPB constructs. Initially, a seven‐factor model based on the IPPAMQ was tested to identify key factors associated with nurses’ behavioral intentions. This model mapped the pathways from the three types of beliefs (behavioral, normative, and control) to attitudes, subjective norms, and perceived behavioral control, ultimately linking them to nurses’ behavioral intentions. Subsequently, an eight‐factor model was examined to assess the framework’s explanatory power with nurses’ self‐reported practices as the primary outcome. Maximum likelihood estimation was adopted for parameter estimation, and path modifications were applied based on theoretically supported modification indices. Model fit was evaluated using RMSEA (≤ 0.080), SRMR (≤ 0.100), CFI (≥ 0.900), TLI (≥ 0.900), and PNFI (≥ 0.500). Because model fit indices may be affected by model complexity and dimensionality [36], overall model adequacy was evaluated based on the collective evidence from multiple indices rather than a single criterion. The strength of relationships and the proportion of variance explained were represented by standardized regression coefficients (*β*) and squared multiple correlations (*R* ^2^).

### 2.6. Ethical and Institutional Approval

This study was reviewed and approved by the Institutional Review Boards (IRBs) of five institutions prior to its commencement (Cardinal Tien Hospital IRB CTH‐112‐3‐5‐031, Dalin Tzu Chi Hospital IRB B11203020, Hualien Tzu Chi Hospital Research Ethics Committee IRB 112–006 B, Mackay Memorial Hospital IRB 23MMHIS357e, and Taichung Tzu Chi Hospital Research Ethics Committee REC113‐44), with one approval applying to three participating institutions. Each participant provided written informed consent.

## 3. Results

Information regarding the participants’ individual characteristics and the distribution of the collected data is presented in Table 1.

**TABLE 1:** Individual characteristics of study participants and data distribution.

Variable	Phase I *n* = 200	Phase II *n* = 308	Total *n* = 508	
	*n* (%)/Mean (SD)	*n* (%)/Mean (SD)	*n* (%)/Mean (SD)	Range Actual (possible)
Age (years)	30.0 (7.5)	29.4 (6.9)	29.6 (7.2)	20–58
20–25 years	69 (34.5)	105 (34.2)	174 (34.3)	
26–30 years	63 (31.5)	101 (32.9)	164 (32.3)	
31–40 years	40 (20.0)	69 (22.5)	109 (21.5)	
> 40 years	28 (14.0)	32 (10.4)	60 (11.8)	
Gender				
Female	176 (88.0)	274 (89.0)	452 (89.0)	
Male	24 (12.0)	34 (11.0)	56 (11.0)	
Educational level				
Diploma or associate degree	31 (15.5)	48 (15.7)	79 (15.6)	
Bachelor degree	167 (83.5)	256 (83.0)	423 (83.2)	
Master degree	2 (1.0)	4 (1.3)	6 (1.2)	
Institution type				
Medical center	60 (30.0)	147 (47.7)	207 (40.7)	
Regional hospital	140 (70.0)	161 (52.3)	301 (59.3)	
Type of ICU				
With surgical patients (surgical, cardiac, neurological, or burn ICUs)	41 (20.5)	146 (47.4)	187 (36.8)	
Without surgical patients (medical, cardiac, or respiratory ICUs)	159 (79.5)	162 (52.6)	321 (63.2)	
Critical care experience (years)	6.8 (6.2)	6.8 (6.1)	6.8 (6.1)	0.25–37.08
< 2 years	48 (24.0)	64 (20.8)	112 (22.0)	
2–5 years	60 (30.0)	105 (34.1)	165 (32.5)	
> 5–10 years	47 (23.5)	72 (23.4)	119 (23.4)	
> 10 years	45 (22.5)	67 (21.8)	112 (22.0)	
Pain assessment training for critically ill adults				
No	92 (46.5)	125 (40.8)	217 (43.1)	
Yes	106 (53.5)	181 (59.2)	287 (56.9)	
Pain management training for critically ill adults				
No	101 (50.8)	141 (46.1)	242 (47.9)	
Yes	98 (49.2)	165 (53.9)	263 (52.1)	
The Behavioral Scale score (5 items)	26.7 (4.8)	27.4 (5.0)	27.1 (4.9)	6–35 (5–35)
The IPPAMQ subscale scores				
Intention (4 items)	23.1 (3.3)	23.2 (3.5)	23.2 (3.4)	12–28 (4–28)
Behavioral beliefs (5 items)	32.0 (3.7)	31.7 (4.1)	31.8 (3.9)	17–35 (5–35)
Normative beliefs (5 items)	28.0 (4.8)	28.3 (5.0)	28.2 (4.9)	9–35 (5–35)
Control beliefs (5 items)	28.6 (4.9)	29.0 (4.4)	28.9 (4.6)	5–35 (5–35)
Attitude toward the behavior (4 items)	20.5 (6.1)	20.5 (6.1)	20.1 (6.1)	4–28 (4–28)
Subjective norm (4 items)	22.9 (3.7)	22.7 (4.1)	22.7 (4.0)	8–28 (4–28)
Perceived behavioral control (3 items)	16.9 (2.8)	17.2 (2.9)	17.1 (2.9)	7–21 (3–21)

Abbreviations: ICU = intensive care unit; IPPAMQ = the Intention to Perform Pain Assessment and Management Questionnaire; SD = standard deviation.

### 3.1. Phase I—Instrument Development and Validation (*n* = 200)

#### 3.1.1. The IPPAMQ

##### 3.1.1.1. Item Quality

The absolute values of skewness (0.30–2.06) and kurtosis (0.05–6.36) for the items in the initial version indicate acceptable normality. All items showed significant differences between the upper and lower 27% groups and had adequate corrected item‐total correlations (0.50–0.86).

##### 3.1.1.2. Construct Validity

CFA tested the initial version of the IPPAMQ against a proposed seven‐factor model. The initial analysis prompted the removal of eight items (one item each from the behavioral beliefs, normative beliefs, attitude toward the behavior, subjective norms, and intention subscales, and three items from the perceived behavioral control subscale). These items were removed because they had factor loadings < 0.50, modification indices of standardized residuals > 4.00, or were semantically similar to other items within the same subscales. According to the recommended thresholds (RMSEA ≤ 0.080, SRMR ≤ 0.100, CFI ≥ 0.900, TLI ≥ 0.900, and PNFI ≥ 0.500), the 30‐item IPPAMQ (Table S4) demonstrated an acceptable model fit (RMSEA = 0.059, SRMR = 0.055, CFI = 0.938, TLI = 0.930, and PNFI = 0.761).

Table 2 presents the results of the analysis for convergent validity. The item factor loadings (0.52–0.93) and the CR values for the seven subscales (0.78–0.93) were adequate. The AVE values for six subscales were adequate (0.58–0.73), with the intention subscale having a suboptimal value (0.48). Table S5 presents the results related to the discriminant validity evaluation. Two squared interfactor correlation values were higher than the AVE value of the intention subscale. We examined interfactor correlations, paying special attention to the two correlations (intention *vs.* normative beliefs and intention *vs.* perceived behavioral control). Subsequently, we obtained satisfactory discriminant validity, as all interfactor correlations were lower than 0.85 (0.18–0.75; *p* < 0.001).

**TABLE 2:** Convergent validity and reliability of the IPPAMQ (*n* = 200).

Total or subscale	Item	Convergent validity			Reliability	
		Standardized factor loading	CR	AVE	Internal consistency: McDonald’s omega (*ω*)	Test–retest *ICC*
Evaluation criteria		≥ 0.50	≥ 0.70	≥ 0.50	≥ 0.70	≥ 0.40

IPPAMQ total (30 items)		—	—	—	0.93	0.81^∗∗∗^

Intention (4 items)	Intention1	0.87	0.78	0.48	0.77	0.48^∗∗^
	Intention2	0.77				0.52^∗∗^
	Intention3	0.52				0.52^∗∗^
	Intention4	0.56				0.48^∗∗^

Behavioral beliefs (5 items)	Bb1	0.90	0.93	0.73	0.92	0.74^∗∗∗^
	Bb2	0.93				0.56^∗∗∗^
	Bb3	0.93				0.64^∗∗∗^
	Bb4	0.72				0.56^∗∗∗^
	Bb5	0.76				0.49^∗∗^

Normative beliefs (5 items)	Nb1	0.72	0.92	0.69	0.91	0.42^∗∗^
	Nb2	0.82				0.47^∗∗^
	Nb3	0.86				0.52^∗∗^
	Nb4	0.83				0.51^∗∗^
	Nb5	0.91				0.68^∗∗∗^

Control beliefs (5 items)	Cb1	0.73	0.91	0.67	0.91	0.52^∗∗^
	Cb2	0.87				0.41^∗^
	Cb3	0.85				0.50^∗∗^
	Cb4	0.87				0.42^∗∗^
	Cb5	0.76				0.46^∗∗^

Attitude toward the behavior (4 items)	Atb1	0.89	0.85	0.58	0.84	0.80^∗∗∗^
	Atb2	0.64				0.49^∗∗^
	Atb3	0.87				0.44^∗∗^
	Atb4	0.61				0.48^∗∗^

Subjective norm (4 items)	Sn1	0.66	0.88	0.65	0.87	0.60^∗∗∗^
	Sn2	0.90				0.57^∗∗∗^
	Sn3	0.92				0.59^∗∗∗^
	Sn4	0.72				0.47^∗∗^

Perceived behavioral control (3 items)	Pbc1	0.75	0.85	0.66	0.85	0.43^∗∗^
	Pbc2	0.83				0.44^∗∗^
	Pbc3	0.85				0.41^∗∗^

Abbreviations: Atb = attitude toward the behavior; AVE = average variance extracted; Bb = behavioral beliefs; Cb = control beliefs; CR = composite reliability; *ICC* = intraclass correlation coefficient; IPPAMQ = the Intention to Perform Pain Assessment and Management Questionnaire; Nb = normative beliefs; Pbc = perceived behavioral control; Sn = subjective norm.

^∗^ *p* < 0.05, ^∗∗^ *p* < 0.01, ^∗∗∗^ *p* < 0.001.

##### 3.1.1.3. Reliability

The internal consistency of the 30‐item IPPAMQ (McDonald’s *ω* = 0.93) and its seven subscales (McDonald’s *ω* = 0.77–0.92) was adequate. The test–retest reliability for the IPPAMQ’s total scale (*ICC* = 0.81, *p* < 0.001) and its 30 items (*ICC* = 0.41–0.80, *p* < 0.050) was adequate (Table 2).

#### 3.1.2. The Behavioral Scale

The absolute values of skewness (0.36–0.74) and kurtosis (0.05–0.59) for the Behavioral Scale demonstrated acceptable normality. Each item effectively discriminated between the upper and lower 27% of respondents (*p* < 0.001) and had adequate corrected item‐total correlations (0.59–0.78). The construct validity of the Behavioral Scale was supported by significant group differences: the Behavioral Scale scores for nurses who had received pain assessment (27.6 *vs.* 26.5; *t* = 2.33, *p* = 0.020) or pain management (27.6 *vs.* 26.6; *t* = 2.42, *p* = 0.016) training and who were employed at ICUs with surgical patients (27.8 *vs.* 26.7; *t* = 2.55, *p* = 0.011) were significantly higher than those for nurses without these characteristics. This scale demonstrated satisfactory internal consistency (McDonald’s *ω* = 0.87) and test–retest reliability for the total scale and individual items (*ICC* = 0.42–0.61, *p* < 0.050).

### 3.2. Phase II—Evaluation of Model Explanatory Power (*n* = 308)

#### 3.2.1. Identifying Factors Associated With Behavioral Intentions

We used data obtained from the 30‐item IPPAMQ to evaluate the proposed seven‐factor model. To improve model fit, two residual covariances were added based on modification indices. According to the recommended thresholds (RMSEA ≤ 0.080, SRMR ≤ 0.100, CFI ≥ 0.900, TLI ≥ 0.900, and PNFI ≥ 0.500), the refined seven‐factor model demonstrated generally acceptable fit indices (RMSEA = 0.075, SRMR = 0.128, CFI = 0.911, TLI = 0.902, and PNFI = 0.785), although the SRMR exceeded the conventional threshold. Within this model, (a) attitude toward the behavior (*β* = 0.13, *p* = 0.010), subjective norm (*β* = 0.16, *p* = 0.014), and perceived behavioral control (*β* = 0.71, *p* < 0.001) were significantly related to behavioral intention, collectively accounting for 65% of the variance in behavioral intention; (b) behavioral beliefs (*β* = 0.33, *p* < 0.001) were significantly correlated with attitude toward the behavior; (c) normative beliefs (*β* = 0.62, *p* < 0.001) were significantly associated with subjective norm; and (d) control beliefs (*β* = 0.72, *p* < 0.001) were significantly linked to perceived behavioral control. The six significant paths are aligned with the TPB’s hypothesized relationships (Figure 2).

**FIGURE 2:**
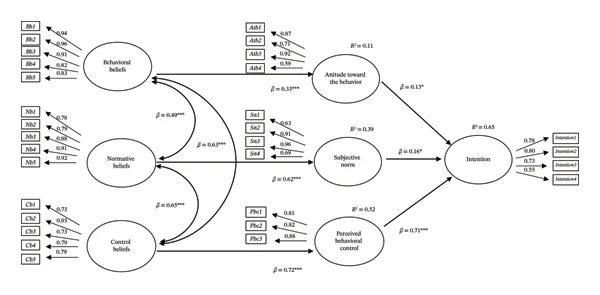
Structural equation modeling of a seven‐factor model based on the IPPAMQ. Abbreviations: Atb = attitude toward the behavior; Bb = behavioral beliefs; Cb = control beliefs; IPPAMQ=Intention to Perform Pain Assessment and Management Questionnaire; Nb = normative beliefs; Pbc = perceived behavioral control; Sn = subjective norm. Notes: To enhance clarity in the presentation, the error terms for all items are omitted; *β* represents the standardized path coefficients; *R* ^2^ represents the squared multiple correlations; ^∗^ *p* < 0.05, ^∗∗^ *p* < 0.01, ^∗∗∗^ *p* < 0.001.

#### 3.2.2. Assessing the Explanatory Power for Nursing Practices

An eight‐factor model integrating the Behavioral Scale with the IPPAMQ was subsequently examined. According to the recommended thresholds (RMSEA ≤ 0.080, SRMR ≤ 0.100, CFI ≥ 0.900, TLI ≥ 0.900, and PNFI ≥ 0.500), the model achieved an acceptable overall model fit (RMSEA = 0.068, CFI = 0.910, TLI = 0.903, PNFI = 0.788) after incorporating two residual covariances based on modification indices. Although the SRMR (0.124) exceeded the conventional threshold, the remaining primary indices consistently supported the model’s overall adequacy. Behavioral intention was significantly associated with behavior (nurses’ pain assessment and management practices; *β* = 0.62, *p* < 0.001), accounting for 47% of the variance. Perceived behavioral control was not associated with behavior. Attitude toward the behavior (*β* = 0.13, *p* = 0.010), subjective norm (*β* = 0.15, *p* = 0.017), and perceived behavioral control (*β* = 0.72, *p* < 0.001) were significantly related to behavioral intention, collectively explaining 65% of the total variance. The three types of beliefs demonstrated significant associations with their corresponding constructs. The seven significant paths support the TPB’s hypothesized relationships (Figure 3).

**FIGURE 3:**
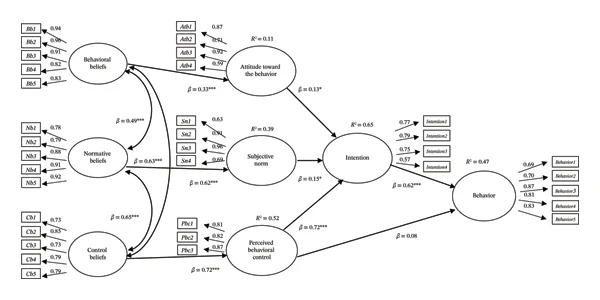
Structural equation modeling of an eight‐factor model based on the IPPAMQ and the Behavioral Scale. Abbreviations: Atb = attitude toward the behavior; Bb = behavioral beliefs; Cb = control beliefs; IPPAMQ=Intention to Perform Pain Assessment and Management Questionnaire; Nb = normative beliefs; Pbc = perceived behavioral control; Sn=subjective norm. Notes: To enhance clarity in the presentation, the error terms for all items are omitted; *β* represents the standardized path coefficients; *R* ^2^ represents the squared multiple correlations; ^∗^ *p* < 0.05, ^∗∗^ *p* < 0.01, ^∗∗∗^ *p* < 0.001.

## 4. Discussion

This study developed and psychometrically tested the IPPAMQ and the Behavioral Scale to establish satisfactory psychometric properties for both instruments. Using the data obtained from these validated instruments, we examined the structural relationships within the TPB framework. Critical care nurses’ attitudes, subjective norms, and perceived behavioral control were significantly associated with their behavioral intentions. The three types of beliefs demonstrated significant associations with their corresponding constructs. Nurses’ intentions were significantly associated with their self‐reported practices, reinforcing the TPB’s conceptual strength in explaining nurses’ clinical practices.

A major methodological strength of this study is the application of SEM (CFA and path analysis). This approach rigorously and precisely models the complex relationships among observed and latent variables while systematically accounting for measurement error [24, 31]. It offers a robust framework for understanding the mechanisms through which TPB constructs are associated with nurses’ behavioral intentions and practices.

The inclusion of a behavioral outcome is another strength, as it allows the exploration of how sociopsychological constructs relate to nurses’ clinical practices. However, nurses may give socially desirable responses to protect their professional identities. Self‐reported measures may capture what nurses intend to do, what they believe they do, or their idealized understanding of institutional guidelines. Therefore, rigorous procedural safeguards (e.g., ensuring complete respondent anonymity, using on‐site sealed collection boxes, and applying validated instruments) were instituted during data collection to mitigate potential measurement biases. Additionally, a single‐factor CFA was conducted to assess the potential threat of common method bias empirically [24, 37]. According to recommended thresholds (RMSEA ≤ 0.080, SRMR ≤ 0.100, CFI ≥ 0.900, TLI ≥ 0.900, and PNFI ≥ 0.500), the analysis yielded poor model fit (RMSEA = 0.153, SRMR = 0.140, CFI = 0.538, TLI = 0.510, and PNFI = 0.478). The CFA results confirmed that a single common method factor failed to pervasively account for variance, suggesting that common method bias is unlikely to threaten the validity of the findings. Nevertheless, combining these theory‐informed instruments with objective clinical metrics in future studies is recommended to cross‐validate the data.

### 4.1. Instrument Development and Validation

The approach of using operational definitions for the TPB constructs adopted in our study and by Youngcharoen et al.’s studies [20, 21] provides essential guidance for item generation. The item analysis results confirmed the item quality of the IPPAMQ and the Behavioral Scale. The clarity, appropriateness, and relevance of the items to their corresponding constructs were further verified through intensive discussions among committee members and careful expert evaluations. Additionally, a pilot study ensured that the items in the developed instruments were comprehensible to the target population.

The satisfactory factor loadings, AVEs, and CRs supported the convergent validity of the IPPAMQ. All interfactor correlation values indicated acceptable discriminant validity, confirming that each subscale measured a distinct construct. The intention subscale had a suboptimal AVE, which may be attributed to the relatively low factor loadings of one scenario‐based item (Intention4). The information provided for this item was static. When interpreting the scenario, nurses might have filled in information that was not provided, leading to variations in their responses. For example, some might assume that the nurse was “too busy.” Others might question whether the patient’s conditions (e.g., level of consciousness) permit effective communication. Furthermore, certain concepts (e.g., subsequent healthcare management) within the item description may be ambiguous and subject to varying interpretations. We recommend future refinements to streamline the scenario description and minimize interpretive variance, because the developed items should ideally serve as pure indicators of a single construct [23].

Significant group differences supported the construct validity of the Behavioral Scale. Consistent with the literature [32, 33], nurses who received related training or worked in ICUs caring for surgical patients reported more favorable practices. This highlights the instrument’s capacity to discriminate between varying levels of clinical expertise and the specific demands of practice environments.

Both the IPPAMQ and the Behavioral Scale exhibited satisfactory internal consistency and stability. The IPPAMQ’s belief subscales showed high McDonald’s *ω* values (0.91–0.92). High internal consistency may indicate some level of item overlap. However, this redundancy can be beneficial in the early stages of instrument development, as it helps establish reliability and enhance construct clarity [23].

### 4.2. Evaluation of Model Explanatory Power

Our findings are consistent with the theoretical structure of the TPB. Model testing indicated that both seven‐ and eight‐factor models demonstrated an acceptable overall fit. Prior research indicates that SRMR values may be influenced by model dimensionality and increase as the number of latent factors increases [36, 38]. Therefore, model fit should be evaluated based on multiple indices rather than a single criterion. In this study, the RMSEA, CFI, and TLI met the recommended standards, supporting the overall adequacy of the proposed models [36, 38]. The elevated SRMR values suggest that the residual discrepancies between the observed and model‐implied covariance structures may persist. Therefore, these findings should be interpreted with caution. Supported by converging fit indices and satisfactory PNFI parsimony, the results collectively support the TPB’s utility in explaining nurses’ behavioral intentions and practices regarding pain assessment and management.

#### 4.2.1. Identifying Factors Associated With Behavioral Intentions

Edwards et al. [19] reported that the TPB model explained 39% of the variance in nurses’ behavioral intentions to administer *pro re nata* (PRN) opioids. Our seven‐factor model accounted for 65% of the variance in behavioral intention and demonstrated greater explanatory power than Edwards et al.‘s findings. The validated IPPAMQ can effectively capture the sociopsychological correlates underlying nurses’ behavioral intentions toward pain assessment and management.

Among the identified factors, perceived behavioral control exhibited the strongest correlation with intention, followed by subjective norm and attitude. This hierarchy differs from previous findings [20], which identified attitude and subjective norm as the primary correlates. However, even with positive attitudes and social support, a lack of perceived capability may hinder the formation of strong intention [20]. Our findings underscore the critical role of nurses’ perceived capability, alongside facilitating and constraining environmental factors. These insights highlight the need for supportive organizational structures in ICUs. The relatively weak association with attitude likely reflects that nearly half of the participants lacked professional training. Thus, targeted educational and training interventions are crucial for fostering positive professional attitudes [6, 39].

In line with previous findings [20], the three types of beliefs (behavioral, normative, and control) demonstrated significant associations with their corresponding constructs. These results indicate that these beliefs are fundamental to nurses’ behavioral intentions to perform pain assessment and management. Specifically, positive evaluations of the necessity and benefits of pain assessment and management fostered favorable attitudes. Simultaneously, expectations from key referents (e.g., nurse leaders, physicians, and colleagues) strengthened subjective norms and facilitated adherence to protocols. For example, when nurses perceive that their leaders expect consistent pain assessment and management to achieve optimal patient outcomes, their normative beliefs are strengthened, thereby reinforcing their sense of professional obligation.

#### 4.2.2. Assessing the Explanatory Power for Nursing Practices

Nurses’ behavioral intentions accounted for 47% of the variance in their self‐reported practices. Prior research among healthcare professionals has shown that intention typically explains 21%–31% of behavioral variance [40, 41]. Our results surpass previous findings and underscore the significant association between critical care nurses’ behavioral intentions and their clinical practices.

Perceived behavioral control was not directly associated with behavior but demonstrated an indirect association through behavioral intention. This finding suggests that the performance of pain assessment and management depends more on deliberate intention than on perceived behavioral control. This interpretation aligns with meta‐analytic evidence: while perceived behavioral control may moderate the intention–behavior relationship [42], behavioral intention shows the most direct association with behavioral enactment [17]. Collectively, these findings support the TPB’s robust explanatory power. Attitudes, subjective norms, and perceived behavioral control are associated with behavioral intentions, which in turn are linked to critical care nurses’ clinical practices.

The eight‐factor model explained nearly half of the variance in nurses’ self‐reported practices. The remaining unexplained variance may be attributed to environmental constraints and organizational factors inherent in critical care settings. High patient acuity, competing clinical priorities, sudden physiological deterioration, staffing shortages, and documentation demands frequently hinder nurses from performing comprehensive pain assessment and management, even though they possess strong behavioral intentions [33, 43]. Previous studies have shown that pain assessment and management are among the most frequently omitted nursing activities in critical care settings [44]. Critical care nurses are often required to prioritize physiological stabilization and urgent interventions. Consequently, these interconnected organizational and environmental barriers are associated with lower adherence to standard practices [44, 45], reflecting an intention–practice gap [17, 42] in which favorable behavioral intentions do not consistently align with clinical practices. To address this limitation, future longitudinal or experimental research should incorporate objective organizational and environmental variables to clarify how these constraints relate to clinical behavior and to examine intervention strategies that foster alignment between behavioral intention and clinical practice.

### 4.3. Limitations

This study has several limitations. First, the cross‐sectional design precludes the establishment of definitive causal relationships among TPB constructs. Longitudinal or experimental research is required to confirm whether changes in key factors (e.g., perceived behavioral control) lead to subsequent changes in behavioral intention or clinical practice. Second, the sampling strategy may limit the generalizability of the findings to other healthcare settings or geographical regions. Third, self‐reported data may introduce recall or social desirability bias, as nurses may align their responses with perceived professional or ethical standards. Consequently, these measures may reflect nurses’ perceived adherence rather than their actual clinical practices, necessitating cautious interpretation. Although the developed instrument provides a theory‐informed measure of behavior, it may overestimate compliance relative to objective data. In this regard, direct observations can capture real‐time behavior but may be affected by the Hawthorne Effect [46], whereas medical record audits provide concrete documentation but may underreport nurses’ practices due to time constraints in clinical settings. Therefore, future research should integrate objective metrics or direct observations alongside self‐reports to mitigate response bias and enhance measurement validity.

### 4.4. Implications for Nursing Management

The development of the IPPAMQ provides nurse managers and policymakers with a practical and theoretically informed instrument for systematic monitoring. These stakeholders can use this instrument to periodically assess sociopsychological barriers, thereby enabling a data‐driven approach to identifying specific areas for tailored interventions. Regarding the generalizability of our findings, the development of the IPPAMQ and the Behavioral Scale was grounded in the TPB and international guidelines. These instruments address core concepts (e.g., pre‐procedural pain assessment and interprofessional communication during handovers) applicable across diverse critical care settings. They offer a viable framework for benchmarking nursing practice and exploring sociopsychological factors associated with nurses’ clinical practices. Furthermore, our findings confirmed the TPB’s explanatory power: attitudes, subjective norms, and perceived behavioral control collectively accounted for a substantial proportion of the variance in nurses’ behavioral intentions, and nurses’ behavioral intentions were significantly associated with their clinical practices. These relationships align with international critical care literature highlighting environmental constraints, workload, and resource availability as globally important factors associated with nursing practice [43, 47]. However, variations in healthcare systems, nurse‐to‐patient staffing ratios, institutional policies, and nursing autonomy across different countries may moderate these sociopsychological pathways. Therefore, cross‐cultural adaptation, further psychometric testing, and replication across diverse international critical care settings are recommended to confirm the global generalizability of this framework.

Our identification of the factors associated with nurses’ behavioral intentions will enable the design of targeted management interventions. The TPB’s substantial explanatory power for nurses’ self‐reported practices underscores the need for multilevel strategies targeting modifiable sociopsychological factors. As perceived behavioral control has emerged as the strongest correlate of behavioral intentions, nurse managers and policymakers must enhance nurses’ perceived capability and address environmental constraints. Priorities should include streamlining clinical workflows, ensuring the accessibility of standardized assessment tools, and institutionalizing evidence‐based protocols. Such structural improvements are essential to reduce operational burdens and bolster clinical autonomy [39, 43]. Nurse managers should conduct regular documentation audits paired with timely, constructive feedback. Such targeted coaching minimizes decisional uncertainty, eliminates procedural barriers, and empowers nurses with the confidence to autonomously execute evidence‐based practices.

To reinforce subjective norms, nurse managers should foster supportive leadership, interprofessional collaboration, and peer support to reduce decisional uncertainty [47, 48]. Nurse managers can establish structured pain reporting during nursing handovers and interdisciplinary rounds. Incorporating pain discussions into routine clinical workflows reinforces shared accountability and signals clear leadership expectations across the healthcare team. To foster positive attitudes toward pain assessment and management, nurse managers could shift from traditional regular lectures to frequent, case‐based learning sessions. Engaging nurses in reflective case discussions helps reframe pain assessment and management from a routine administrative burden into a vital patient‐centered intervention. Targeted continuing education is also essential to bridge competency gaps and align professional attitudes with best practices [6, 39], thereby fostering clinical practices that reflect positive behavioral intentions.

## 5. Conclusion

This study successfully achieved its aims by developing and validating the IPPAMQ and the Behavioral Scale, offering psychometrically robust instruments tailored to the TPB framework. The substantial explanatory power of the seven‐factor model supports the IPPAMQ as a valuable tool for assessing the sociopsychological factors underlying nurses’ behavioral intentions. The TPB is an effective framework for identifying the factors associated with behavioral intentions and explaining self‐reported practices in pain assessment and management. The results highlight that perceived behavioral control was associated with behavioral intentions, and behavioral intentions were associated with critical care nurses’ clinical enactment. This study underscores the need to foster supportive work environments and enhance professional training. By focusing on nurses’ perceived capabilities and addressing normative barriers, nurse managers and policymakers can more effectively foster the alignment between intentions and clinical practices, ultimately enhancing pain assessment and management for adult ICU patients.

## Author Contributions

Zhi‐Han Cai contributed to the preparation of the material, data curation, investigation, data analysis, and the drafting of the manuscript and secured resources. Tsung‐Cheng Hsieh performed the data analysis, contributed to the manuscript draft and validation, and provided resources and supervision. Li‐Hwa Lin was responsible for material preparation and data curation, further contributed to the manuscript draft, and provided resources. Hui‐Chun Chung was responsible for data curation, investigation, and providing resources. Sophia Huey‐Lan Hu was responsible for material preparation and provided resources and supervision. Yi‐Shan Hung, Yi‐Ching Lin, Mei‐Yen Lu, Chia‐Hui Li, and Ying‐Shan Chen were responsible for material preparation, data curation, and investigation. Yuan‐Mei Liao contributed to the preparation of the material, data curation, investigation, data analysis, drafting of the manuscript, secured resources, supervision, and funding acquisition. All authors contributed to the study’s conceptualization, critically reviewed and revised this manuscript, and approved the final version.

## Funding

This study was supported by the Higher Education Sprout Project by the Ministry of Education (MOE) in Taiwan (grant number 114DN23).

## Disclosure

The funder was not involved in the writing, editing, approval, or decision to publish the manuscript.

## Conflicts of Interest

The authors declare no conflicts of interest.

## Supporting Information

Additional supporting information can be found online in the Supporting Information section.

## Supporting information


**Supporting Information 1** Table S1. Overview of existing instruments measuring nurses’ pain assessment and management practices for adult patients, structured around four domains (study purpose, instrument content, measured TPB constructs, and psychometric testing results). Table S2. Theoretical definitions of the TPB constructs and operational definitions used in the current study. Table S3. The five Behavioral Scale items developed to reflect core clinical pain assessment and management practices aligned with standard practice guidelines. Table S4. The 30 IPPAMQ items developed based on the TPB framework, covering three types of beliefs (behavioral, normative, and control), attitude, subjective norm, perceived behavioral control, and behavioral intention. Table S5. Information on the discriminant validity of the 30‐item IPPAMQ, including average variance extracted (AVE) estimates, interfactor correlations, and squared interfactor correlations.

**Supporting Information 2** STROBE Checklist‐20260727. R2.

## Data Availability

The data supporting the findings of this study are available within the article’s supporting materials. Additional datasets are available from the corresponding author, upon reasonable request, owing to privacy and ethical restrictions.
