# Supplementary material for: Factors Associated With Behavioral Intentions Toward Pain Assessment and Management Among Critical Care Nurses: A Theory‐Based Instrument Validation and Model Testing Study
Source: J Nurs Manag. 2026 Oct 26;2026:7115245. doi: 10.1155/jonm/7115245 (PMC13696997; doi:10.1155/jonm/7115245)
Supplement: Supplementary file 1 [file JONM-2026-7115245-s002.docx]

**Supplementary Materials**

Table S1. Summary of the study purposes, instrument content, content measured corresponding to the TPB constructs, and psychometric testing results of the identified instruments

| Authors (Year/Country) | Study purpose | Instrument content  /Utilization of a theoretical framework | Content measured corresponding to the TPB constructs | Psychometric tests | |
| --- | --- | --- | --- | --- | --- |
| -Instrument |  |  |  | Internal consistency reliability (Cronbach α) | Validity |
| Edwards et al. (2001/Canada)  - The Pain Management Survey (~60 items, including intention with 3 items; response: 5- and 7-point Likert scales) | Assessing nurses’ intention and predictors of behaviors in relation to administering opioids to relieve pain in hospitalized patients | -Direct attitude  -Indirect (belief-based) attitude (corresponding beliefs)  -Subjective norm (normative beliefs)  -Indirect control (control beliefs)  -Direct control  -Intention  /The TPB | -Beliefs (behavioral, normative, and control)  -Attitude toward the behavior  -Subjective norm  -Perceived behavioral control  -Intention | 0.53-0.79 | -- |
| Rose et al.  (2011/Canada)  -Survey of Pain Assessment and Management for the Critically-Ill (~36 items; response: yes *vs.* no, multiple-choice, a 5-point Likert scale, and open-ended) | Investigating critical care nurses’ practices and knowledge related to pain assessment and management for critically ill adults, able and unable to self-report | -Current pain assessment and management practices for critically ill patients, able and unable to self-report  -Perceived importance of pain assessment  -Perceived relevance of behavioral indicators used as descriptors by validated pain assessment tools  -Enablers and barriers to effective pain assessment, documentation, and management  -Pain beliefs  -Education on pain  /Nil | -Beliefs (behavioral)  -Attitude toward the behavior  -Perceived behavioral control  -Behavior | -- | Face and content validity, and comprehensibility were assessed by experts |

Table S1. Summary of the study purposes, instrument content, content measured corresponding to the TPB constructs, and psychometric testing results of the identified instruments (continued)

| Authors (Year/Country) | Study purpose | Instrument content  /Utilization of a theoretical framework | Content measured corresponding to the TPB constructs | Psychometric tests | |
| --- | --- | --- | --- | --- | --- |
| -Instrument |  |  |  | Internal consistency reliability (Cronbach α) | Validity |
| Youngcharoen et al.  (2016 & 2017/Thailand)  -PAQ and PMQ (~60 items, including intentions with 4 items; response: a 7-point Likert scale) | -Determine the relationships among nurses’ beliefs, attitudes, perceived behavioral control, intentions, and nurses’ pain management for hospitalized postoperative elderly (2016)  -Describe nurses’ beliefs regarding pain assessment and management for hospitalized postoperative elderly (2017)  -For different nurse groups (with different experience duration, institution type, and training received on pain management), present item analysis of the TPB constructs and compare the TPB constructs across different nurse groups (2017) | -Behavioral beliefs  -Normative beliefs  -Control beliefs  -Attitude  -Perceived norm  -Perceived behavioral control  -Intention  -Behaviors (case study vignettes)  /The TPB | -Beliefs (behavioral, normative, and control)  -Attitude toward the behavior  -Subjective norm  -Perceived behavioral control  -Intention  -Behavior | PAQ: 0.61-0.92  PMQ: 0.73-0.98  (for 7 subscales: behavioral beliefs, normative beliefs, control beliefs, attitude, perceived subjective norm, perceived behavioral control, and intention) (2016) | Content validity was assessed by nurses for all subscales (2016) |

Table S1. Summary of the study purposes, instrument content, content measured corresponding to the TPB constructs, and psychometric testing results of the identified instruments (continued)

| Authors (Year/Country) | Study purpose | Instrument content  /Utilization of a theoretical framework | Content measured corresponding to the TPB constructs | Psychometric tests | |
| --- | --- | --- | --- | --- | --- |
| -Instrument |  |  |  | Internal consistency reliability (Cronbach α) | Validity |
| Asman et al.  (2019/Israel)  -A structured questionnaire (~33 items; response: 5- and 7-point Likert scales) | -Examining critical care nurses’ knowledge of behaviors indicating pain in mechanically ventilated patients  -Examining the effects of nurses’ self-perceived nurse-physician collaboration on the adequacy of department pain management | -Nurse’s knowledge of behaviors indicating pain in mechanically ventilated patients  -Nurse-physician self-perceived collaboration and nurse satisfaction with care decisions  -Perceived adequacy of department pain management  /Nil | -Subjective norm  -Perceived behavioral control | 0.82-0.94 | Face validity and comprehensibility were assessed by nurses |
| Sweity et al. (2022/Palestine)  -A structured questionnaire (~38 items; response: true *vs.* false, yes *vs.* no, and multiple-choice) | -Assessing critical care nurses’ knowledge, attitudes, and practices regarding pain management  -Determining possible obstacles to effective pain management | -Nurses’ knowledge of pain for critical care patients  -Nurses’ attitudes toward pain management for critical care patients  -Perceived challenges in caring for critical care patients  -Pain assessment documentation practices for critical care patients  /Nil | -Attitude toward the behavior  -Perceived behavioral control  -Behavior | 0.70 for the knowledge scale | Face and content validity were assessed by experts |

Abbreviations: PAQ=Pain assessment questionnaire; PMQ=Pain management questionnaire; TPB=Theory of Planned Behavior.

*Note*: The contents listed in the column “Contents measured corresponding to the TPB constructs” represent the instruments comprised of items potentially related to the TPB constructs.

Table S2. The operational definitions of the constructs involved in the Behavioral Scale and the IPPAMQ

| Construct | Theory of Planned Behavior (TPB) | Operational definition |
| --- | --- | --- |
| Behavior | Behavior refers to the actual performance of the target action. | Critical care nurses’ self-reported practices in pain assessment and management. |
| Intention | Intention is considered to be the immediate antecedent of a behavior. | The degree to which critical care nurses are prepared to adhere to updated practice guidelines to perform adequate pain assessment and management. |
| Behavioral beliefs | Behavioral beliefs are individuals’ expectations about the outcomes of a behavior. | Critical care nurses’ subjective opinions about the necessity and potential consequences, both positive and negative, for performing pain assessment and management. |
| Normative beliefs | Normative beliefs reflect individuals’ perceptions of others’ expectations. | The concerns critical care nurses have regarding the likelihood that their nurse leaders, physicians, or colleagues approve or disapprove of their adequate practices in pain assessment and management. |
| Control beliefs | Control beliefs pertain to individuals’ opinions on the factors that may facilitate or hinder the execution of a behavior. | Critical care nurses’ perceptions of the factors that may either facilitate or hinder their capability to perform adequate pain assessment and management. |
| Attitude toward the behavior | Attitudes toward a behavior represent individuals’ positive or negative evaluations of a behavior. | Critical care nurses’ overall subjective evaluations for performing pain assessment and management are positively or negatively valued. |
| Subjective norm | Subjective norm reflects individuals’ perceived social pressures. | Critical care nurses’ perceived pressure from nurse leaders, physicians, or colleagues to perform or not to perform adequate pain assessment and management. |
| Perceived behavioral control | Perceived behavioral control reflects individuals’ perceptions of how easy or difficult it is to perform a behavior. | Critical care nurses’ perceptions of their capability and control over adhering to updated practice guidelines and performing adequate pain assessment and management, considering both internal and external factors such as personal competence and the resources available in the work environment. |

Abbreviation: IPPAMQ=Intention to Perform Pain Assessment and Management Questionnaire.

Table S3. The five-item Behavioral Scale

*Note*: “Patients” refer to critically ill patients, and “nurses” refer to critical care nurses.

The following questions contain 7 options. After reading each question, please select the number (and corresponding option) that best reflects your personal experience or opinion based on your current practices. Responses range from “1 = Never” to “7 = Always.”

| Items/Options |  | | | | | | | | |
| --- | --- | --- | --- | --- | --- | --- | --- | --- | --- |
|  | Never | | | Sometimes | | | Always | | |
| ***Behavior: Behavior1-Behavior5*** |  |  |  | |  |  | |  |  |
| 1. How often do you assess pain and provide pain relief medication that meets the needs of patients before performing procedures such as repositioning, suctioning, or wound care? | 1 | 2 | 3 | | 4 | 5 | | 6 | 7 |
| 2. For patients in stable condition who are able to self-report, how often do you document pain assessment results and provide appropriate pain relief management every 4 hours? | 1 | 2 | 3 | | 4 | 5 | | 6 | 7 |
| 3. For patients in stable condition who are unable to self-report, how often do you use a suitable pain assessment scale? | 1 | 2 | 3 | | 4 | 5 | | 6 | 7 |
| 4. How often do you and your colleagues discuss pain assessment scores and pain relief management received for patients unable to self-report during nursing handovers? | 1 | 2 | 3 | | 4 | 5 | | 6 | 7 |
| 5. If patients do not achieve optimal pain control, how often would you review the appropriateness of the implemented pain relief interventions (e.g., pain-related medical orders)? | 1 | 2 | 3 | | 4 | 5 | | 6 | 7 |

*Note*: This English version scale is translated from the traditional Chinese-language version.

Table S4. The 30-item Intention to Perform Pain Assessment and Management Questionnaire (IPPAMQ)

*Note*: “Patients” refer to critically ill patients, and “nurses” refer to critical care nurses.

The following questions contain 7 options. After reading each question, please select the number (and corresponding option) that best reflects your personal experience or opinion based on your current practices. Responses range from “1 = Strongly disagree” to “7 = Strongly agree.”

| Items/Options |  | | | | | | | | | |
| --- | --- | --- | --- | --- | --- | --- | --- | --- | --- | --- |
|  | Strongly disagree | | |  | | | Strongly agree | | | |
| ***Behavioral beliefs: Bb1-Bb5*** *(this line can be omitted during the data collection process)* | | | | | | | | | | |
| 1. Regardless of patients’ ability to self-report their pain, nurses are responsible for ensuring they receive suitable pain assessment and management. | 1 | 2 | 3 | | 4 | 5 | | 6 | 7 |  |
| 2. For patients able or unable to self-report, nurses should adhere to clinical healthcare standards to assess pain and provide adequate pain relief management. | 1 | 2 | 3 | | 4 | 5 | | 6 | 7 |  |
| 3. To provide effective pain assessment and management, nurses must learn to use suitable pain assessment tools and be familiar with clinical healthcare standards and procedures related to pain assessment and management. | 1 | 2 | 3 | | 4 | 5 | | 6 | 7 |  |
| 4. Nurses need to be familiar with pain-related behaviors exhibited by patients unable to self-report in order to identify their pain. | 1 | 2 | 3 | | 4 | 5 | | 6 | 7 |  |
| 5. I believe that nurses following clinical healthcare standards for pain assessment and management promote effective patient pain control and improve overall patient healthcare. | 1 | 2 | 3 | | 4 | 5 | | 6 | 7 |  |
| ***Attitude toward the behavior: Atb1-Atb4*** *(this line can be omitted during the data collection process)* | | | | | | | | | | |
| 6. Pharmacological and non-pharmacological interventions provided by nurses (e.g., listening to music, employing relaxation techniques) help alleviate the pain experienced by patients. | 1 | 2 | 3 | | 4 | 5 | | 6 | 7 |  |
| 7. Nurses should not solely rely on observing changes in patients’ physiological parameters (e.g., increased respiratory/heart rate, elevated blood pressure) to identify their pain. | 1 | 2 | 3 | | 4 | 5 | | 6 | 7 |  |
| 8. I believe that performing pain assessment and management is an important nursing task. | 1 | 2 | 3 | | 4 | 5 | | 6 | 7 |  |
| 9. For patients unable to self-report, I believe that nurses need to be open to learning and applying suitable pain assessment tools and pain management. | 1 | 2 | 3 | | 4 | 5 | | 6 | 7 |  |

Table S4. The 30-item Intention to Perform Pain Assessment and Management Questionnaire (IPPAMQ; continued)

*Note*: “Patients” refer to critically ill patients, and “nurses” refer to critical care nurses.

The following questions contain 7 options. After reading each question, please select the number (and corresponding option) that best reflects your personal experience or opinion based on your current practices. Responses range from “1 = Strongly disagree” to “7 = Strongly agree.”

| Items/Options |  | | | | | | | | | |
| --- | --- | --- | --- | --- | --- | --- | --- | --- | --- | --- |
|  | Strongly disagree | | |  | | | Strongly agree | | | |
| ***Subjective norm: Sn1-Sn4*** *(this line can be omitted during the data collection process)* | | | | | | | | | | |
| 10. Regardless of whether patients can express their pain, I feel that the physicians, nurse leaders, and nursing colleagues in my unit expect me to follow clinical healthcare standards in assessing and managing patients’ pain. | 1 | 2 | 3 | | 4 | 5 | | 6 | 7 |  |
| 11. I feel that the physicians and nurse leaders in my unit support and agree with me in prioritizing patients’ pain assessment and management as essential healthcare tasks. | 1 | 2 | 3 | | 4 | 5 | | 6 | 7 |  |
| 12. I feel that my nursing colleagues support and agree with me in prioritizing patients’ pain assessment and management as essential healthcare tasks. | 1 | 2 | 3 | | 4 | 5 | | 6 | 7 |  |
| 13. I feel that all nursing colleagues in my unit follow the institution’s pain clinical healthcare standards in assessing and managing patients’ pain. | 1 | 2 | 3 | | 4 | 5 | | 6 | 7 |  |
| ***Perceived behavioral control: Pbc1-Pbc3*** *(this line can be omitted during the data collection process)* | | | | | | | | | | |
| 14. I feel that I have sufficient competence to identify pain-related behaviors exhibited by patients unable to self-report, such as changes in facial expression or restlessness. | 1 | 2 | 3 | | 4 | 5 | | 6 | 7 |  |
| 15. The pain assessment tools and clinical healthcare standards available in our institution and units enable me to provide suitable pain assessment and management for patients able and unable to self-report. | 1 | 2 | 3 | | 4 | 5 | | 6 | 7 |  |
| 16. Based on the pain-related medical orders prescribed by physicians, I can provide suitable pain assessment and pain relief management for patients able and unable to self-report. | 1 | 2 | 3 | | 4 | 5 | | 6 | 7 |  |

Table S4. The 30-item Intention to Perform Pain Assessment and Management Questionnaire (IPPAMQ; continued)

*Note*: “Patients” refer to critically ill patients, and “nurses” refer to critical care nurses.

The following questions contain 7 options. After reading each question, please select the number (and corresponding option) that best reflects your personal experience or opinion based on your current practices. Range from “1 = Extremely unlikely” to “7 = Extremely likely.”

| Items/Options |  | | | | | | | | |
| --- | --- | --- | --- | --- | --- | --- | --- | --- | --- |
|  | Extremely unlikely | | |  | | | Extremely likely | | |
| ***Normative beliefs: Nb1-Nb5*** *(this line can be omitted during the data collection process)* | | | | | | | | | |
| 17. To what extent do you prioritize the expectations of unit physicians when performing pain assessment and management? | 1 | 2 | 3 | | 4 | 5 | | 6 | 7 |
| 18. How likely do you think your nurse leaders expect nurses to follow clinical healthcare standards when performing pain assessment and management for ICU patients? | 1 | 2 | 3 | | 4 | 5 | | 6 | 7 |
| 19. To what extent do you prioritize the expectations of nurse leaders when performing pain assessment and management? | 1 | 2 | 3 | | 4 | 5 | | 6 | 7 |
| 20. How likely do you think your nursing colleagues expect nurses to follow clinical healthcare standards in performing patient pain assessment and management? | 1 | 2 | 3 | | 4 | 5 | | 6 | 7 |
| 21. To what extent do you prioritize the expectations of nursing colleagues when performing pain assessment and management? | 1 | 2 | 3 | | 4 | 5 | | 6 | 7 |
| ***Control beliefs: Cb1-Cb5*** *(this line can be omitted during the data collection process)* | | | | | | | | | |
| 22. How likely do you think a nurse’s competence in pain assessment and management would influence their adherence to clinical healthcare standards in performing patient pain assessment and management? | 1 | 2 | 3 | | 4 | 5 | | 6 | 7 |
| 23. How likely do you think the pain assessment and management education and training nurses receive would influence their adherence to clinical healthcare standards in performing patient pain assessment and management? | 1 | 2 | 3 | | 4 | 5 | | 6 | 7 |
| 24. How likely do you think the documentation sections for pain assessment and management, or the alert functions within the electronic health record system, influence nurses’ adherence to clinical healthcare standards when performing patient pain assessment and management? | 1 | 2 | 3 | | 4 | 5 | | 6 | 7 |
| 25. How likely do you think the pain assessment tools available at the institutions would influence nurses’ adherence to clinical healthcare standards in performing patient pain assessment and management? | 1 | 2 | 3 | | 4 | 5 | | 6 | 7 |
| 26. How likely do you think physicians’ precise prescriptions for pain relief medications and sufficient analgesics would influence nurses’ adherence to clinical healthcare standards in performing patient pain assessment and management? | 1 | 2 | 3 | | 4 | 5 | | 6 | 7 |

Table S4. The 30-item Intention to Perform Pain Assessment and Management Questionnaire (IPPAMQ; continued)

*Note*: “Patients” refer to critically ill patients, and “nurses” refer to critical care nurses.

The following questions contain 7 options. After reading each question, please select the number (and corresponding option) that best reflects your personal experience or opinion based on your practices over the next three months. Range from “1 = Extremely unlikely” to “7 = Extremely likely.”

| Items/Options |  | | | | | | | | |
| --- | --- | --- | --- | --- | --- | --- | --- | --- | --- |
|  | Extremely unlikely | |  | | | Extremely likely | | | |
| ***Intention: Intention1-Intention4*** *(this line can be omitted during the data collection process)* | | | | | | | | |  |
| 27. I tend to assess the pain experienced by patients according to clinical healthcare standards, implement pain relief management based on the pain assessment results and clinical practice guidelines, and ensure timely completion of relevant documentation. | 1 | 2 | 3 | 4 | 5 | | 6 | 7 |  |
| 28. How likely are you and your colleagues to discuss patients’ pain assessment scores and pain relief management received during nursing handovers? | 1 | 2 | 3 | 4 | 5 | | 6 | 7 |  |
| 29. How likely are you to proactively provide suitable pain relief interventions before performing medical or nursing procedures that may cause pain (e.g., repositioning, removing drainage tubes)? | 1 | 2 | 3 | 4 | 5 | | 6 | 7 |  |
| 30. Mr. Wang is a 60-year-old ICU patient with an endotracheal tube and ventilator support. Mr. Wang, with a GCS score of E_3_V_E_M_3_, is unable to express himself. While assisting Mr. Wang with a chest tube dressing change, the nurse noticed that he was frowning, withdrawing his limbs, and experiencing muscle rigidity. In response, the nurse increased the dosage of his fentanyl pump to help alleviate his pain. Twenty minutes later, the nurse observed that Mr. Wang was no longer frowning or experiencing muscle rigidity.  Given this scenario, if you were the nurse responsible for Mr. Wang’s healthcare, how likely would you be to reassess his pain within 1 hour and provide subsequent healthcare management based on the pain assessment results? | 1 | 2 | 3 | 4 | 5 | | 6 | 7 |  |

Abbreviations: Atb=Attitude toward the behavior; Bb=Behavioral beliefs; Cb=Control beliefs; GCS=Glasgow Coma Scale; Nb=Normative beliefs; Pbc=Perceived behavioral control; Sn=Subjective norm.

*Note*: This English version questionnaire is translated from the traditional Chinese-language version.

Table S5. Discriminant validity of the 30-item IPPAMQ

| Subscales | Intention | Behavioral beliefs | Normative beliefs | Control beliefs | Attitude toward the behavior | Subjective norm | Perceived behavioral control |
| --- | --- | --- | --- | --- | --- | --- | --- |
| Number of items | 4 items | 5 items | 5 items | 5 items | 4 items | 4 items | 3 items |
| Intention | **0.48** | 0.62^***^ | 0.71^***^ | 0.58^***^ | 0.20^***^ | 0.64^***^ | 0.72^***^ |
| Behavioral beliefs | 0.39 | **0.73** | 0.49^***^ | 0.45^***^ | 0.21^***^ | 0.61^***^ | 0.59^***^ |
| Normative beliefs | 0.50^#^ | 0.24 | **0.69** | 0.64^***^ | 0.20^***^ | 0.65^***^ | 0.59^***^ |
| Control beliefs | 0.34 | 0.20 | 0.41 | **0.67** | 0.18^**^ | 0.44^***^ | 0.45^***^ |
| Attitude toward the behavior | 0.04 | 0.04 | 0.04 | 0.03 | **0.58** | 0.28^***^ | 0.28^***^ |
| Subjective norm | 0.41 | 0.38 | 0.42 | 0.20 | 0.08 | **0.65** | 0.75^***^ |
| Perceived behavioral control | 0.51^#^ | 0.35 | 0.35 | 0.20 | 0.08 | 0.56 | **0.66** |

Abbreviation: IPPAMQ=Intention to Perform Pain Assessment and Management Questionnaire.

*Notes*: Average Variance Extracted (AVE) estimates are shown on the bold diagonal. Inter-factor correlations are reported above the diagonal, and squared inter-factor correlations are reported below the diagonal. ^#^Two squared inter-factor correlation values (intention *vs.* normative beliefs, and intention *vs.* perceived behavioral control) were higher than the AVE value of the intention subscale; ^*^*p*<0.05, ^**^*p*<0.01, ^***^*p*<0.001.
